# Harnessing public health with “metaverse” technology

**DOI:** 10.3389/fpubh.2022.1030574

**Published:** 2022-12-01

**Authors:** Sudip Bhattacharya, Saurabh Varshney, Shailesh Tripathi

**Affiliations:** ^1^Department of Community and Family Medicine, All India Institute of Medical Sciences (AIIMS), Deoghar, Jharkhand, India; ^2^All India Institute of Medical Sciences (AIIMS), Deoghar, Jharkhand, India; ^3^Rajendra Institute of Medical Sciences (RIMS), Ranchi, Jharkhand, India

**Keywords:** metaverse, augmented reality, virtual reality, digital health, blockchain, metaverse in medicine, public health

## Introduction

India's economy has been growing and its people's living standards have been improving, but this has happened at the same time as industrialization, urbanization, an aging population, changes in lifestyles, and a wide range of diseases that create new problems for healthcare (1). The Ayushman Bharat Digital Mission (ABDM) wants to make sure that the country's integrated digital health infrastructure can keep running. Through digital highways, it would close the distance between the various healthcare ecosystem players. The goal of ABDM is to build a national digital health ecosystem that is effective, affordable, accessible, inclusive, safe, and supports universal health coverage. This ecosystem should also offer a wide range of data, information, and infrastructure services by using open, interoperable, standards-based digital systems and making sure that personal health information is safe, private, and confidential (2). It is also important for India's efforts to take part in global health governance and keep its promises under the UN 2030 Agenda for Sustainable Development. Building a healthy India means putting more focus on preventive care and making more efforts to improve healthcare systems, improve organization and execution, encourage scientific and technical innovation, and create digital health information services.

## Problem statement

Small rural hospitals in India often don't have access to modern medical equipment (called “low equipment coverage”). Also, local doctors often don't have much clinical experience (called “low technological competence”), and patients often don't understand health issues (called “low patient satisfaction”). This is because hospitals and regions have different amounts of healthcare resources (3). Because of these three problems, many patients prefer to go to major hospitals for better diagnosis and treatment (the “Three Low Areas”). This causes problems with registration and administration, which we call the “Two Difficult Areas.” The large number of people from rural areas who go to hospitals in cities limits how much time each specialist can spend with each patient. This means that services in the “Four Limitation Areas” of preventive healthcare, disease management, and rehabilitation are limited. To carry out the Healthy India Plan, it is very important to change the way things are now (4). A timely answer to these issues has emerged with the rise of the Medical Internet of Things (MIoT), since it offers strong technical assistance. In medicine, the Internet of Things, which can be helped by Augmented Reality (AR) and Virtual Reality (VR) glasses, is called the “metaverse.” The metaverse is developing; it has great promise in healthcare by fusing technologies like Artificial Intelligence (AI), VR, AR, Internet of Medical Devices (IoMD), Web 3.0, intelligent cloud, edge, and quantum computing with robots to give healthcare new directions. According to the current Medical Internet of Things (MIoT) theory, doctors in large hospitals (called “Cloud Experts”) and doctors in smaller hospitals (called “Terminal Doctors”) can work together to make graded diagnosis and treatment more accurate and effective. They can also help study and develop related technologies to improve graded diagnosis and treatment (1). Experts in the cloud are not always and everywhere available to devote time to popular scientific education or to deliver expert lectures (4). According to the current Medical Internet of Things (MIoT) theory, doctors in large hospitals (called “Cloud Experts”) and doctors in smaller hospitals (called “Terminal Doctors”) can work together to make graded diagnosis and treatment more accurate and effective. They can also support the study and development of related technologies to improve graded diagnosis and treatment (4). However, the following problems still exist in practical practice: (1) Cloud Experts are not always and everywhere available to devote time to popular scientific education or to deliver expert lectures. (2) The Cloud Experts aren't always available to help the Terminal Doctors figure out what's wrong and how to treat it. (3) During clinical trials, the principal investigators are not always there to watch over the study and lead the team. (4) There is still a significant amount of non-standard diagnosis and treatment, with characteristics of a handicraft workshop, because there is no real-time quality control in all situations. (5) The circumstances. It is the limits of Internet technology that prevent contact between Cloud Experts and Terminal Doctors engaged in graded diagnosis and therapy from constantly being facilitated. Because of this, MIoT-based digital platforms need to keep getting better, especially in the areas of communication and interaction between humans and computers, as well as the integration and linking of the virtual and real worlds, Ultimately, to realize “complex problems are simplified; simple problems are digitized; digital problems are programmed; and program problems are systematized” (5).

## Medicial uses of metaverse

Telepresence, digital twinning, and blockchain are three significant technology advancements that are converging in the metaverse and have the potential to have an individual influence on healthcare. As our ability to map and understand each person's genes improves, digital twinning will become possible (6). Blockchains are essentially decentralized, encrypted databases that allow for the safe storage of data. Health records are frequently kept on centralized systems, which increases the danger of data theft (7). Additionally, physicians and nurses have discovered that using a phone call or video conference allows them to identify numerous minor conditions—which account for most of their caseload—more swiftly and effectively (8). Thanks to telemedicine consultations, particularly those utilizing virtual reality, patients are no longer restricted to being treated by specific physicians based on their physical location. Doctors and specialists are using VR to teach other doctors and medical personnel. But when they work together, they might develop whole new ways to offer treatment that could save costs and significantly enhance patient outcomes (8, 9).

### Growing use of virtual reality in medical education

The Metaverse is a great place for medical staff to learn because it immerses the user. For medical education, the Metaverse is going in the following directions: tactile haptic controls are used in simulation training to give students a close-up view of a surgical operation, and video recordings of study sessions can be used to make sure that exams are correct. Interactive training materials that improve understanding of fundamental principles, such as virtual reality (VR) experiences that immerse learners within the human body and offer a 360-degree perspective of a patient's condition (Table 1).

**Table 1:** Uses of metaverse.

**Field**	**Application**	**Examples**
**Medicine/psychiatry**	Telemedicine consultation Patient data security through blockchain	VR game called EndeavorRX by Akili Interactive became the first-ever prescription-strength video game approved by the FDA to treat ADHD in children
**Medical education**	Interactive training of healthcare workers Anatomical training for the medical students	Currently, 8chili is a Medtech startup working on the AR/VR platform for the Healthcare Industry and building surgical training
**Surgery/facial reconstructive surgery**	Surgeons will see the body's vital signs, photos, medical history, and any other information in real time	Medical startup Accuvein launched a map of the patient's veins onto the skin In 2020, a surgeon at John Hopkins conducted the first spinal neuro-navigation procedure conducted by an AR headset called xvision by startup Augmedic Arbrea Labs is working on the face simulation tool
**Digital therapeutic applications**	Nurses can visit patients in the Metaverse and do daily check-ups and consultations with the use of remote monitoring NCD prevention through social prescribing and social tokens Differentiation between benign and malignant lesion	
**Radiology**	Increased viewing and manipulation of images View moving pictures with more information to aid in the diagnosis of illness or injury	Image reconstruction
**Medical wearables**	Respiration monitoring	In COVID-19, COPD
**Health promotion**	It is done through virtual Yoga training	Alo Yoga in collaboration with Roblox announced the opening of the virtual ‘Alo Sanctuary,' an immersive wellness space for yoga and meditation

### The metaverse's digital therapeutic applications

There are already indications that the Metaverse will offer new medicinal application possibilities. The field of digital therapies is growing quickly, with uses like cognitive therapy, support groups, psychiatric evaluations, rehabilitation, and even physical treatment in the metaverse, made possible with the help of haptic sensors and VR and AR technology (4). The COVID-19 pandemic has accelerated the uptake of digital technologies. As an example, nurses can attend and consult patients using metaverse, without the chance of spreading infections to one another, like influenza, ebola, and zika virus. Additionally, for NCD prevention and management, metaverse can engage patients by behavioral modification such as stress reduction, weight loss interventions, which can eventually lead to increment in quality adjusted life years and reduction in disability adjusted life years. Social prescribing and social tokens system through metaverse have a potential to prevent NCDs in the future. In resource poor settings, like India, the management of chronic obstructive pulmonary disease (COPD) and obstructive sleep apnea-hypopnea syndrome (OSAHS), is quite challenging, which can be overcome by the use of metaverse technology (10). In 2014, the Chinese Alliance Against Lung Cancer set up the first sub-center for finding and treating pulmonary nodules at Zhongshan Hospital Fudan University. Specialists from the respiratory, thoracic, and radiology departments work closely with the terminal at the pulmonary nodule special outpatient clinic. Professor Bai's team used Internet of Things (IoT) medicine to accurately tell people who had benign or cancerous lung nodules what was wrong with them. In the 6 years leading up to 2019, there were 16,400 lung nodule surgeries. Pathological analysis showed that 60.8% of these surgeries were for early-stage lung cancer (4). These early-stage lung cancer patients have great outcomes for the country, the patients, and the doctors because they do not need chemotherapy, radiation, targeted therapy, or immunotherapy after surgery (4).

### Surgical procedures using augmented reality

The Metaverse is expected to be a useful tool that will help surgeons do difficult operations and improve patient care. Now, surgeons use cutting-edge technology like robots to do operations (8–11). The Metaverse has already created a lot of enthusiasm and predictions about its potential in almost every aspect of surgery with the help of other doctors and specialists, surgeons will work together in a virtual operating room. Surgeons will see the body's vital signs, photos, medical history, and any other information they need to know about the patient. New technology that works with the Metaverse can give quick test results in real time to help patients do better. At first, the Metaverse will be used slowly because clinical trials are needed to find out if it is a useful tool for surgery. We expect the Metaverse to get more and more attention as its use grows, just like how robot-assisted surgery is getting more and more attention.

### Metaverse in radiology

**Figure 1:**
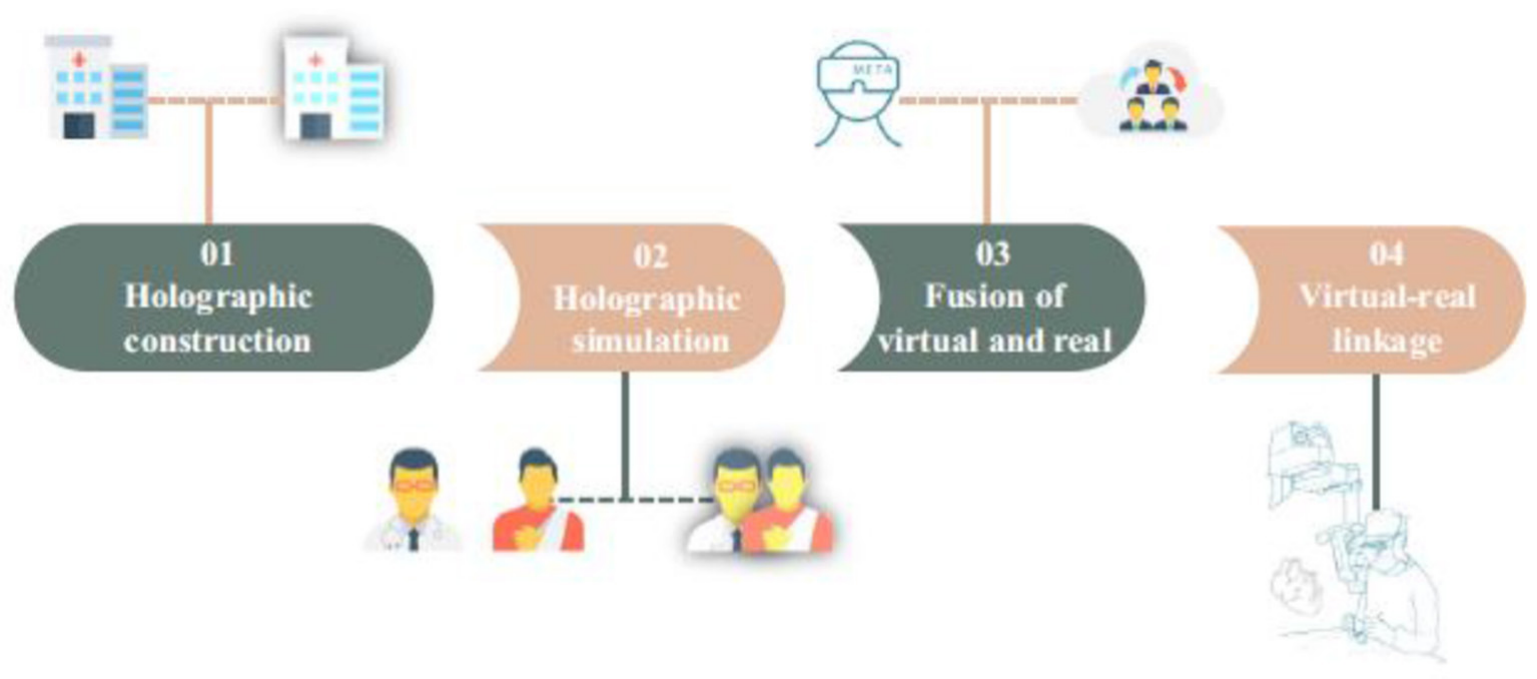
The four stages of metaverse in medicine.

With the immersive visual capabilities of the metaverse, radiological imaging is one area of medicine that will benefit, opening new possibilities like:

1. Increased viewing and manipulation of images.
2. View moving pictures with more information to aid in the diagnosis of illness or injury.
3. It encourages strong cooperation between doctors while working with 3D medical pictures (Figure 1).

### The metaverse's use of medical wearables

The metaverse will make it easier for both patients and doctors to use medical devices that are worn on the body. Devices, such as COPD monitors, can notify emergency personnel and caregivers to do wellness checks if a patient, for instance, has a COPD flare-up. Also, it is expected that wearables will give doctors better data, which will improve virtual consultations in the Metaverse by using data gathered in real time.

### The metaverse's challenges for healthcare and patient care

#### Getting people into the metaverse

What strategies will Meta and other tech firms use to get users into the metaverse? It is up to the users of a new technology to establish momentum in the market. This raises a lot of concerns regarding the metaverse's reliability and usefulness. Would older people who are less tech-aware be able to access the metaverse and receive the best treatment, for instance? Or would people value appointments in the metaverse equally to in-person encounters? To answer these questions, we can say that-although, in addition to younger generations, digital seniors, who are aged 60 years and above had learned how to stay connected within the digital ecosystems with children and grandchildren while remained abroad. Meanwhile, some digital seniors are also using wearable devices and this is how the market of metaverse is expanding. As an example, US-based company *Rendever* is supplying VR equipment to the digital seniors and giving them the opportunity to enjoy immersive virtual entertainment and travel experiences while staying at home.

#### New health insurance portability and accountability regulations

Making sure that patients' private information, especially PHI and ePHI, is safe and secure will make it harder for people to use the metaverse. Since health insurance portability and accountability (HIPAA) rules have changed because of the growth of telemedicine and mobile device integration, laws will need to be changed to consider the metaverse's wealth of information.

#### Security and privacy issues in the metaverse

The safety of patient data in the metaverse presents another difficulty. By putting the metaverse into healthcare, which is often a high-value and vulnerable target for cybercriminals, a whole new set of problems needs to be solved. With more patient data available, privacy is also a constant worry. It will take time for patients to decide if they feel comfortable communicating with and interacting with the metaverse on a large scale, but early findings are encouraging.

#### Issues with interoperability

Interoperability is one of the most difficult parts of the current healthcare paradigm when it comes to implementing new technologies. Adding a new factor, like the metaverse, can have a big and unpredictable effect on how easy it is to move devices across different platforms and networks. Adoption of the metaverse will be slow and might even be bad if the entire healthcare industry doesn't quickly improve data and communication standards.

#### High technology costs

It takes a big infrastructure to run the metaverse's potential in healthcare to its fullest extent. The startup may be out of reach for many doctors and hospitals because it uses uninterruptible 5G and high-tech hardware like eyewear, sensors, and other wearables. This has an impact on the patients as well, who might need equipment to follow treatment plans and supervision. Would insurance companies be willing to pay for devices that give access to the metaverse, or would patients have to pay for them?

#### New business models that benefit all parties

All healthcare businesses will need to come up with a new business model that includes the metaverse if they want to keep making money. Just a few things to think about in terms of how the metaverse may affect healthcare are listed below:

1. Insurance companies may decide to treat payments for virtual health visits differently from payments for in-person appointments.
2. Time spent in the metaverse may need to be recorded in malpractice cases for liability reasons.
3. For sessions particular to the metaverse, commercial payers will require specialized medical codes.
4. Vendors of software will need to make their products and associated hardware metaverse-compatible.
5. The question of whether doctors may recommend medications and/or therapies virtually must be seriously discussed.

But there will be problems that will make people pay close attention to the many new and changing dimensions of the metaverse. By thinking about these possible risks, the health care industry will set higher standards for this new and immersive technology. The immersive visuals of the metaverse will make the field of radiology imaging better, which will open up new powers. There are also certain difficulties that the metaverse in healthcare presents. Healthcare is often a high-value and easy-to-hack target, and for the metaverse to be fully used in healthcare, a large infrastructure is needed. To our knowledge, the top three healthcare organizations engaged in metaverse work are:

- Latus Healthcare: This organization is creating a “virtual hospital.” It consists of a virtual reality hospital setting, where first treatments will emphasize physiotherapy services (12).
- iMining: The Decentral and Metaverse's first hospital foundation ever to be established (13).
- Apollo Hospitals: To facilitate involvement in the metaverse, the Apollo Hospital Group has launched a novel partnership with “8chili Inc” (14).
- All India Institutes of Medical Sciences (AIIMS), New Delhi implemented new digital surgery technology from ImmersiveTouch on January 13, 2022.

#### Expert consensus on metaverse in medicine

Recently, Bai et al. have proposed a definition of the Metaverse in Medicine as the medical Internet of Things (MIoT) facilitated using AR and/or VR glasses. A multi-disciplinary panel of doctors and IT experts from Asia, the United States, and Europe analyzed published articles regarding expert consensus on the Medical Internet of Things, with reference to study results in the field of metaverse technology. Research findings suggested that it is feasible to implement the three basic functions of the MIoT, namely, comprehensive perception, reliable transmission, and intelligent processing, by applying a metaverse platform, which is composed of AR and VR glasses and the MIoT system, and integrated with the technologies of holographic construction, holographic emulation, virtuality-reality integration, and virtuality-reality interconnection (15).

## Conclusion

To sum up, the metaverse is an immersive technology that is growing quickly and has a lot of potential to improve patient care across the board in the healthcare industry. It's great that the idea of the metaverse has been spread around the world. It could be a solution to most health problems and will help people in India propose and build on the idea of the metaverse in medicine.

## Author contributions

All authors listed have made a substantial, direct, and intellectual contribution to the work and approved it for publication.

## Conflict of interest

The authors declare that the research was conducted in the absence of any commercial or financial relationships that could be construed as a potential conflict of interest.

## Publisher's note

All claims expressed in this article are solely those of the authors and do not necessarily represent those of their affiliated organizations, or those of the publisher, the editors and the reviewers. Any product that may be evaluated in this article, or claim that may be made by its manufacturer, is not guaranteed or endorsed by the publisher.

## References

[1] The History of Economic Development in India since Independence - Association for Asian Studies. Available online at: https://www.asianstudies.org/publications/eaa/archives/the-history-of-economic-development-in-india-since-independence/ (accessed August 9, 2022).

[2] NHA | Official website Ayushman Bharat Digital Mission. Available online at: https://ndhm.gov.in/abdm (accessed August 9, 2022).

[3] India still struggles with rural doctor shortages - The Lancet. Available online at: https://www.thelancet.com/journals/lancet/article/PIIS0140-6736(15)01231-3/fulltext (accessed August 9, 2022). 10.1016/S0140-6736(15)01231-3 26700521

[4] Yang D Zhou J Song Y Sun M Bai C. Metaverse in medicine. Clin eHealth. (2022) 5:39–43. 10.1016/j.ceh.2022.04.002

[5] Yesmin T Carter MW Gladman AS. Internet of things in healthcare for patient safety: an empirical study. BMC Health Serv Res. (2022) 22:278. 10.1186/s12913-022-07620-3 35232433 PMC8889732

[6] United, Nations. The Impact of Digital Technologies. United Nations. Available online at: https://www.un.org/en/un75/impact-digital-technologies (accessed August 9, 2022).

[7] Bhattacharya S Singh A Hossain MM. Strengthening public health surveillance through blockchain technology. AIMS Public Health. (2019) 6:326–33. 10.3934/publichealth.2019.3.326 31637281 PMC6779606

[8] Bhattacharya S Pradhan KB Bashar MA Tripathi S Semwal J Marzo RR. Artificial intelligence enabled healthcare: a hype, hope or harm. J Fam Med Prim Care. (2019) 8:3461–4. 10.4103/jfmpc.jfmpc_155_19 31803636 PMC6881935

[9] Bhattacharya S. Artificial intelligence, human intelligence, and the future of public health[J]. AIMS Public Health. (2022) 9:644–50. 10.3934/publichealth.2022045 PMC9807415 36636147

[10] Sun M Xie L Liu Y Li K Jiang B Lu Y. The metaverse in current digital medicine. Clin eHealth. (2022) 5:52–7. 10.1016/j.ceh.2022.07.002

[11] Bhattacharya S Hossain MY Juyal R Sharma N Pradhan KB Singh A. Role of public health ethics for responsible use of artificial intelligence technologies. Indian J Community Med. (2021) 46:178–81. 10.4103/ijcm.IJCM_62_20 34321721 PMC8281853

[12] Latus Health. Latus Health. Available online at: https://latushealth.co.uk/ (accessed August 9, 2022).

[13] iMining- Productivity through Real-time Data/IOT. iMining. Available online at: https://www.imining.tech (accessed August 9, 2022).

[14] In In a first-of-its-kind initiative in the healthcare industry Apollo Hospitals collaborates with 8chili Inc to enter the Metaverse. Apollo Hospitals. Available online at: https://www.apollohospitals.com/apollo-in-the-news/in-a-first-of-its-kind-initiative-in-the-healthcare-industry-apollo-hospitals-collaborates-with-8chili-inc-to-enter-the-metaverse/ (accessed August 9, 2022).

[15] Yang D Zhou J Chen R Song Y Song Z Zhang X. Expert consensus on the metaverse in medicine. Clin eHealth. (2022) 5:1–9. 10.1016/j.ceh.2022.02.001

